# Greater usage and positive mood change for users of a dynamic VR app before and after the COVID-19 pandemic onset

**DOI:** 10.3389/fpsyg.2024.1278207

**Published:** 2024-02-27

**Authors:** Jessica Housand, Allen Cornelius, Karen E. Shackleford

**Affiliations:** ^1^School of Psychology, Fielding Graduate University, Santa Barbara, CA, United States; ^2^Department of Public Health, Johns Hopkins University, Baltimore, MD, United States

**Keywords:** virtual reality, stress, mood, VR, interactivity, presence, pandemic, COVID-19

## Abstract

Americans reported an increase in stress during the novel coronavirus disease 2019 (COVID-19). Virtual reality (VR) apps have been shown to distract users from stressors in the environment, but little is known about the efficacy of specific content features to reduce stress or improve mood for consumer users during a pandemic. The present study investigated secondary archival data to explore how mood and usage behavior changed before and after the onset of COVID-19 for consumer users of a VR app with dynamic, interactive content. Study findings indicate that the COVID-19 pandemic had significant effects on user behavior and mood. Users created more accounts and used app content more often during the pandemic, while reporting increased negative mood states. This suggests that users were motivated to use the content to cope with pandemic stressors. Users also experienced a greater positive mood change after using the content during the pandemic than before, which implies that elements related to the VR app content met users’ psychological needs. Passive content with less interactivity resulted in a greater positive mood state after the COVID-19 onset, likely related to its capacity to reduce stress, facilitate restoration, and improve persistent affective states in stressful environments. This study offers a vital window into how consumer users respond to psychosocial pandemic stressors outside of a controlled environment as well as the prospective for VR app content to serve as a valuable mental health intervention during similar stressful events.

## 1 Introduction

As the sense of normalcy from pre-pandemic life returns, significant impacts on mental health remain. U.S. deaths from the virus continue to climb to over 1.1 million deaths (Centers for Disease Control and Prevention, 2023). The need for effective mental health tools has increased as Americans reported prolonged and acute stress during the height of the pandemic. In fact, the American Psychological Association (APA) has warned that we are facing a national mental health crisis that could have serious repercussions for our health and society in the years to come (American Psychological Association, 2020; Centers for Disease Control and Prevention, 2020). The demand for useful interventions to support mental health during psychosocial pandemic-like stressors has advanced the exploration of media content with the capacity to relieve psychological stress, such as virtual reality (VR) software applications, also referred to as VR apps. VR technology involves the use of a headset that allows a user to interact with app content via a simulated three-dimensional (3D) virtual world. One method to investigate whether specific VR app content may improve stress during a pandemic environment is to evaluate trends of usage behavior and reported mood for home users of those apps during COVID-19.

Because stress can have markedly negative long-term health consequences (Segerstrom and Miller, 2004; Leger et al., 2018; Song et al., 2019), determining how Americans have managed this stress at home and what methods have been most effective in the pandemic context can shed light on future interventions. The growth of technology for communication in a post-COVID-19 onset world has contributed to a rising population of consumer users with greater access to devices and apps promoted as stress management tools for use at home. More immersive devices, such as VR headsets and associated apps, are becoming increasingly common as well, along with virtual content designed to promote stress reduction.

While the literature is rich with studies exploring how VR may be an effective method for distraction in and out of the healthcare setting (Hoffman et al., 2014; Small et al., 2015; Burns-Nadera et al., 2017; Boylan et al., 2018; Khadra et al., 2018; Piskorz and Czub, 2018; Soltani et al., 2018; Hoffman et al., 2019; Spiegel et al., 2019), it is more limited on the usage behavior and efficacy of VR apps with unique content features on psychological stress and anxiety reduction, excluding the growing literature on mindfulness and meditative practices (Seabrook et al., 2020; Mazgelyte et al., 2021; Drigas et al., 2022; Kelly et al., 2022; Mitsea et al., 2022, 2023). Moreover, the psychological effects of these content features and user app use have not been adequately investigated in the consumer user population during a pandemic setting.

This study utilized a quasi-experimental repeated measures design with consumer archival data to examine whether the COVID-19 pandemic was associated with an increase in psychological stress, associated behaviors, and affective states for users of different types of VR app content. The study explored whether users increased app usage for certain content when compared to their usage prior to the pandemic. The study also investigated whether usage led to better mood score outcomes reported by these users. Finally, the study examined whether users during the pandemic who experienced additional interactive content features reported more positive mood scores than with the lower levels of interactive content. Consequently, this research provides a unique window into consumer VR app user behavior, motivations, coping mechanisms, interactive content feature effects, and mood outcomes outside of a controlled environment and in the context of a larger psychosocial stressor event. It also provides important implications related to the efficacy of VR app content as an intervention for stress, mood regulation, and wellness for users at home during pandemic or related stressful contexts.

The VR app used for this study is the TRIPP app, a wellness platform with unique, dynamic content designed to reduce anxiety, increase focus, and increase feelings of calm. TRIPP app content experiences include various virtual environments with dynamic visual and audio features that are referred to in this study as worldscapes. When a user engages in a worldscape with the VR app, this is indicated as a run.

This paper will discuss the literature review and theoretical frameworks underlying the psychological impacts of VR content, associated user behavior, and the unique features of VR that may enable stress reduction. Then, the paper will review study methodology and findings before discussing COVID-19 as a predictor for usage behavior and mood as well as interactions with mood and interactivity level. The paper will consider limitations, conclusions, and close with suggestions for future studies.

## 2 Literature review

### 2.1 Stress regulation and VR content

Psychological convention on stress regulation implies that consumer users at home during the pandemic are likely to seek out the most effective methods to manage stress available. Coping with stress consists of the “cognitive and behavioral efforts to master, reduce, or tolerate the internal and/or external demands that are created by the stressful transaction” and involves both emotional regulation and problem management (Folkman, 1984, p. 843). Established theories around arousal, mood management, stress and coping, as well as self-regulation all suggest that stress reduction can be facilitated through stimuli or resources in the environment that provide the appropriate level of arousal and/or emotional regulation (Berlyne, 1971; Folkman, 1984; Zillmann, 1988a,b; Bandura, 1991).

Berlyne (1971) implies that individuals will seek out the appropriate stimuli in an effort to obtain a desired state of arousal. Individuals generally prefer an intermediate level of arousal and find this to be a more pleasant experience (Berlyne, 1971). Therefore, those who are bored or under-stimulated and more isolated at home will select media devices and content that increase arousal, while those who are stressed or over-aroused in the same environment will opt for relaxing media stimuli to regulate arousal levels.

VR device content can serve as a feature in the environment which promotes a sense of control and positive distraction, two of the psychological needs that should facilitate a user’s ability to cope with stressors (Ulrich et al., 1991; Ulrich, 2001). Perceived control can be influenced via interaction, while VR content with dynamic, naturalistic elements is expected to enhance this effect because of the inherently pleasing properties of nature and its restorative effects (Gerber et al., 2017; Tanja-Dijkstra et al., 2018). The ability to experience content evocative of nature is likely to be especially important when supportive outdoor recreational activities are limited.

Positive distractions, such as VR app content, accessible in the home environment may influence the experience of positive feelings and distraction from negative stimuli. The level of interactivity the user has with the virtual environment as well as the level of control the user has over conducting tasks and manipulating objects in that environment should influence perceived control, which can be expected to increase the sense of presence (Witmer and Singer, 1998). Users are encouraged to choose content that produces the greatest sense of presence and distraction from outside environmental stressors, including content features with the highest level of interactivity.

The uses and gratifications (U&G) theory (Katz et al., 1973; Rubin, 2002), along with the compensatory internet use theory (Kardefelt-Winther, 2014), suggest that users will continue to be motivated to choose and use devices and features of those devices if certain psychological needs are met. Needs motivate behavioral outcomes when users select media content that is expected to offer gratifications or expected gains (Rauschnabel, 2018). These needs include cognitive, informational, tension-release, diversion, affective or aesthetic experience needs, and/or to regulate negative emotion (Katz et al., 1974; Kardefelt-Winther, 2014; Rauschnabel, 2018).

Many resources and coping options were limited during some of the most stressful months of the pandemic. However, when users have access to a resource in the environment that facilitates positive distraction from stressors, reduces arousal in high stimulation environments, influences an intermediate level of arousal, provides a coping resource in the environment that helps to promote emotional regulation, and meets certain psychological needs, a reduction in stress can reasonably be expected. Users should choose to engage in VR app content when environmental stress increases in order to regulate this stress.

Therefore, for users of an app delivered by a VR headset with dynamic content, we predict:

*H1*: Users will use content more frequently during the Pandemic than Pre-Pandemic.

### 2.2 Mood and usage behavior

The relationship between stress and mood is complex. Research shows that stress hormones, such as cortisol and norepinephrine, have associations with low mood as well as anxiety (Drigas and Mitsea, 2020). In contrast, serotonin and oxytocin are linked with positive mood states (Drigas and Mitsea, 2020). Furthermore, psychosocial stressors can modify sensitivity to oxytocin through alterations in brain region connectivity (Fan et al., 2015). Mood is of great interest for investigating whether usage of specific content on VR devices can influence a more persistent affective state than initial, short-lived emotional responses to stimuli.

Affective responses to pandemic stress may best be measured by mood because it reflects a user’s appraisal of his or her relationship with the environment and the larger overarching factors in life (Lazarus, 1991; Morris, 1992; Russell, 2003, 2005; Ekkekakis, 2013). While there is some argument that mood lacks a direct object, other research supports the notion that moods often reflect a more generalized or overview outlook on the greater issues in one’s life rather than an adaptation to a particular stimulus (Lazarus, 1991; Morris, 1992; Russell, 2003, 2005; Ekkekakis, 2013). For these reasons, user mood changes in response to usage of VR content are expected to be the most representative of how a user is feeling, not just about the situation in the immediate environment, but also the overarching domain. Lower mood levels reported before using an app may accurately reflect the longer-term effect that COVID-19 has had on a user’s life outlook, while higher mood scores after using an app would then suggest that the app content positively influenced this overarching mood state.

Mood management theory posits that a user’s current emotional state will influence, either directly or indirectly, what media content that user assesses will optimize mood (Zillmann, 2000). This includes efforts to promote emotional regulation, such as reducing negative mood and maintaining positive mood (Zillmann, 2000). Users are likely to choose content designed to reduce stress and boost mood, since users may be motivated, in part, to satisfy affective or aesthetic needs and improve negative affect (Kardefelt-Winther, 2014; Rauschnabel, 2018). Because emotions and mood are associated with self-regulation, engaging in behavior with VR app content that induces positive affect will likely encourage continued engagement with this feature of the environment (Fredrickson, 2001). In this view, users would be innately motivated to engage in VR content that meets their affective goals based on their feelings related to the pandemic when they are likely to feel stressed and experience low mood.

Because mood is expected to be sensitive to longer term psychosocial stressors present in a user’s environment, and users are innately motivated to choose media content that will potentially reduce stress and boost negative mood, we predict that for users of an app delivered by a VR headset with dynamic content:

*H2*: Users will report lower mood scores at the start of content runs during the Pandemic than Pre-Pandemic.

### 2.3 Immersive VR: presence and interactivity

Characteristics associated with VR app content, such as presence, immersion, and interactivity play a role in relieving user stress through various mechanisms and interrelationships. The experience of presence is important for distracting from unpleasant stimuli and may mediate the restorative effects of VR nature-based content (De Kort et al., 2006). Presence may mirror similar components as that of restoration (Lessiter et al., 2001; Schubert et al., 2001; Freeman, 2004; De Kort et al., 2006). According to Mitsea et al. (2023), VR content minimizes cognitive load, mental fatigue, and increases processing capacity. Several studies have shown that fun ratings for VR positively correlate with sense of presence and pain reduction (Hoffman et al., 2006, 2008; Maani et al., 2011). Immersive factors, such as interactivity may also influence a greater experience of presence, as will increased control over the VR environment, particularly with greater ability to manipulate elements in that environment.

Previous studies have shown that interactivity plays a critical role in how effective device content may be as a distraction from stressors and unpleasant stimuli in the greater environment (Sil et al., 2014; Boylan et al., 2018). For example, one study showed that pain tolerance was improved with engagement in more active media, such as a video game or VR world, than simply watching video clips (Boylan et al., 2018). Another study found that the interactivity of a video game itself on a Nintendo Wii console was enough to effectively distract children from pain and improve pain tolerance (Sil et al., 2014). Furthermore, complexity that requires greater task interaction and additional challenges may be an even more effective distractor (Piskorz and Czub, 2018).

VR content may not only distract a user’s attention but also direct the user’s attention toward targeted goals or behaviors (Mitsea et al., 2023). Digital game-based tasks that challenge the user to overcome obstacles through attention or observation offer intrinsic or external rewards and reinforcement. They can improve self-efficacy, mental and emotional well-being as well as self-regulation (Mitsea et al., 2023). These games can also enhance positive emotions, satisfy psychological needs, and contribute to feelings of control (Mitsea et al., 2023). Presence, interaction, and immersion are thought to further enhance the VR gaming experience so that it is less stressful, more enjoyable, and meaningful (Mitsea et al., 2023). For example, VR games can positively impact feelings of autonomy, competence, need satisfaction, and greater positive feelings than traditional game displays (Mitsea et al., 2023). VR app content with gaming elements also contributes to self-motivation and drives performance improvement (Mitsea et al., 2023).

The passive content in this study more likely utilizes positive distraction and restoration as a means of improving stress and mood. While this VR content also includes high levels of presence and immersion, it requires less interactivity than the active content. The active content involves a basic VR game, which entails task challenges and, based on past literature, should result in superior stress reduction during increased pandemic stress. Principles around coping, perceived control, arousal optimization, gratification, and mood regulation merged with a high interactivity level for the active content reinforce this assumption. Furthermore, if the pandemic causes a greater mood decrement overall, then a greater change in mood from before a user engages in the active content vs. after can be anticipated.

Consequently, for users of an app delivered by a VR headset with dynamic content, we predict:

*H3*: Interactivity level (active vs. passive) will interact with Time (Pre-Pandemic vs. Pandemic) such that mood score change will be higher for active content during but not before the Pandemic.

## 3 Methods

### 3.1 Participants

Participants for this study were taken from anonymous user archival data via the TRIPP app for users that met specific criteria, such as geographic location, completed (pre post) survey data, and data presence across the full time series. The number of U.S.-based users meeting criteria for the study included 14,653 accounts. The gender composition of these users was approximately 53.7% male, 38.2% female, and another 7.3% had no answer. Exclusion criteria included any users that did not provide complete survey data, those who reported as users outside of the U.S., or those who did not have data available for the time period needed for the assessed dates.

### 3.2 Measures

#### 3.2.1 Mood

The Mood score was measured via a quantitative mood survey that appeared at the beginning and end of each worldscape run. Users could select a number from 1 to 10 (1 = Poor, 10 = Excellent) to represent their perceived mood, resulting in a Mood Scale score. The mood scale used by TRIPP was developed by a team at the National Mental Health Innovation Center and appears to have good face validity because it is expected to provide an appropriate measure of subjective mood scores from the individual user perspective across time. Because data was collected via archival data, time-related and carryover effects could not be controlled for. However, to ensure high levels of internal reliability, individual variation was controlled for with the within-subjects design and repeated measures.

#### 3.2.2 Usage

Usage behavior was measured across time by exploring several variables derived from the data that indicated when account users accessed specific TRIPP content. This included the number and frequency of worldscape runs by week and month. The number of accounts and whether an account had active users who had used the content within a certain time frame were also examined to determine usage behavior.

#### 3.2.3 Time frame

Pre-Pandemic was defined as between 7/24/2019 to 3/9/2020, while Pandemic was defined as a period of time between 3/15/2020 to 9/1/2020. The dates for the Pre-Pandemic and Pandemic event for all hypotheses were separated by an interval of 5 days from March 10–14, which included the March 11 date when COVID-19 was declared a pandemic by the WHO and the March 13, 2020 date when COVID-19 was declared a national emergency in the U.S. This time frame was selected to allow for a one-day period before COVID-19 was declared a pandemic by the WHO and 1 day after COVID-19 was declared a national emergency in the U.S. to account for variations in when users may have become aware of pandemic status. Therefore, data from these interval dates were generally not included in hypotheses analysis.

### 3.3 VR experience

The user experience with the TRIPP app involved the use of a head-mounted display to fully immerse the user into a simulated environment with visual and sound elements. The headset utilized a head-tracking system that allowed natural movement for the user within the VR world. The TRIPP app content in this study also had interactive capabilities as well, so that the user could take action to affect the VR environment. The user could choose to interact with the environment by fixating one’s gaze on an interactive object in the worldscape. At the beginning of the worldscape run, the user is asked to rate their mood. After they complete the VR experience, the user is asked to rate their mood again.

#### 3.3.1 Active vs. passive

The interactivity level of the content was defined as either active or passive. Passive content has a lower level of interactive content designed to promote relaxation, reduce psychological stress, and improve mood. Active content has a higher level of interactivity designed to promote focus, reduce psychological stress, and improve mood. It also includes an interactive arcade-style game with tasks that must be completed to move forward with the experience in the TRIPP app.

For this study, the VR app passive content consisted of content with a lower level of interactivity designed to promote relaxation, reduce psychological stress, and improve mood. This VR experience featured natural and organic scenes with movement, color and shape changes, interest points, and gentle music. TRIPP reports that average run time for these passive worldscapes is 13.77 min.

The active content is defined as content with a higher level of interactivity designed to promote focus, reduce psychological stress, and improve mood, featuring similar content as the passive worldscapes but with the addition of psychedelic visuals and an interactive arcade-style game with tasks that must be completed to move forward with the experience in the app. The tasks may include using a randomly colored disc shooter to clear a row of three discs of the same color before the remaining discs reach the end of a line or making contact with coins between obstacles. Average run time for these worldscapes is reported by TRIPP to be 8.08 min.

**Figure 1:**
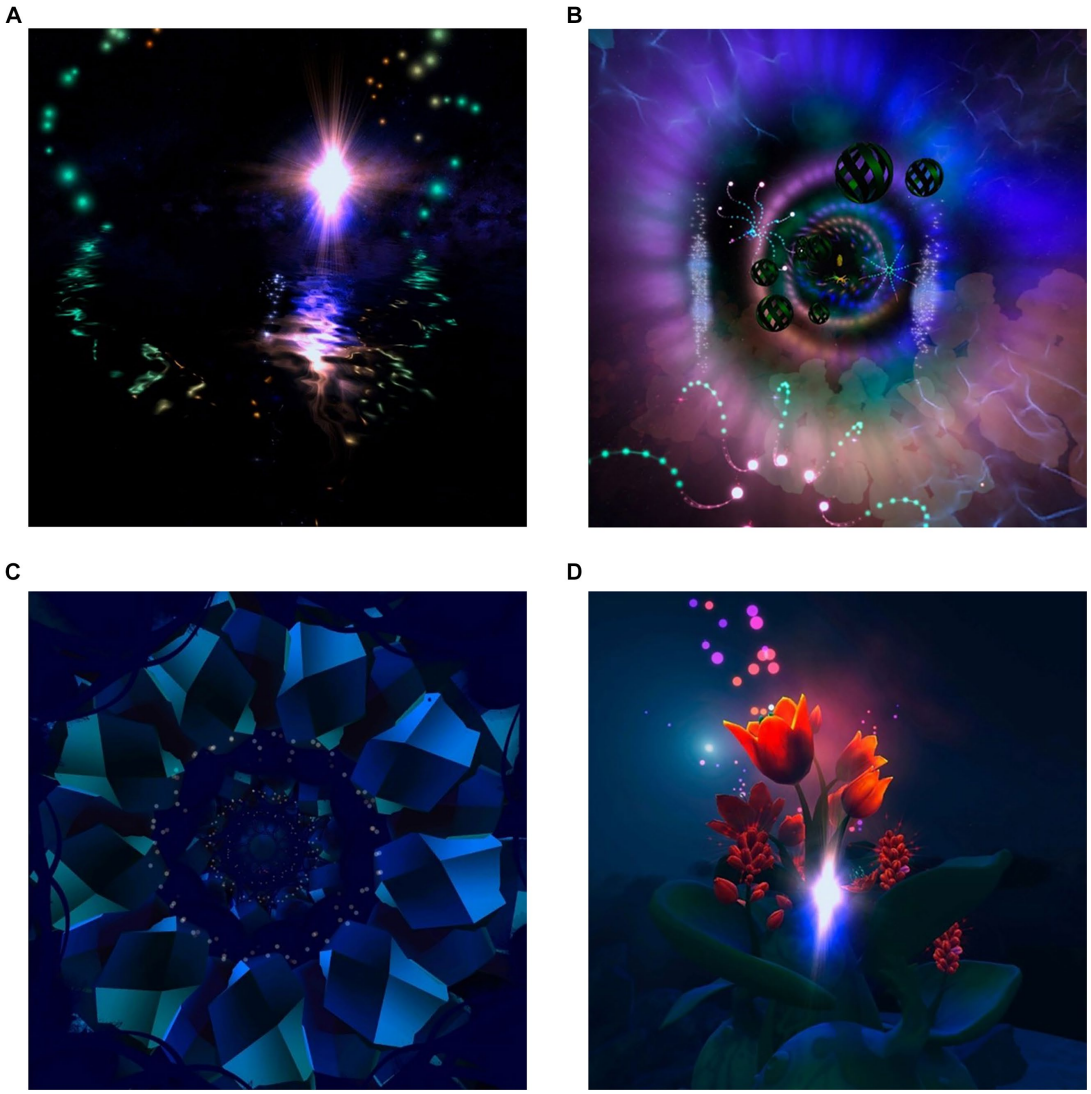
Dynamic, nature-based TRIPP content **(A–D)**. Reproduced with permission from TRIPP, Inc, www.tripp.com.

**Figure 2:**
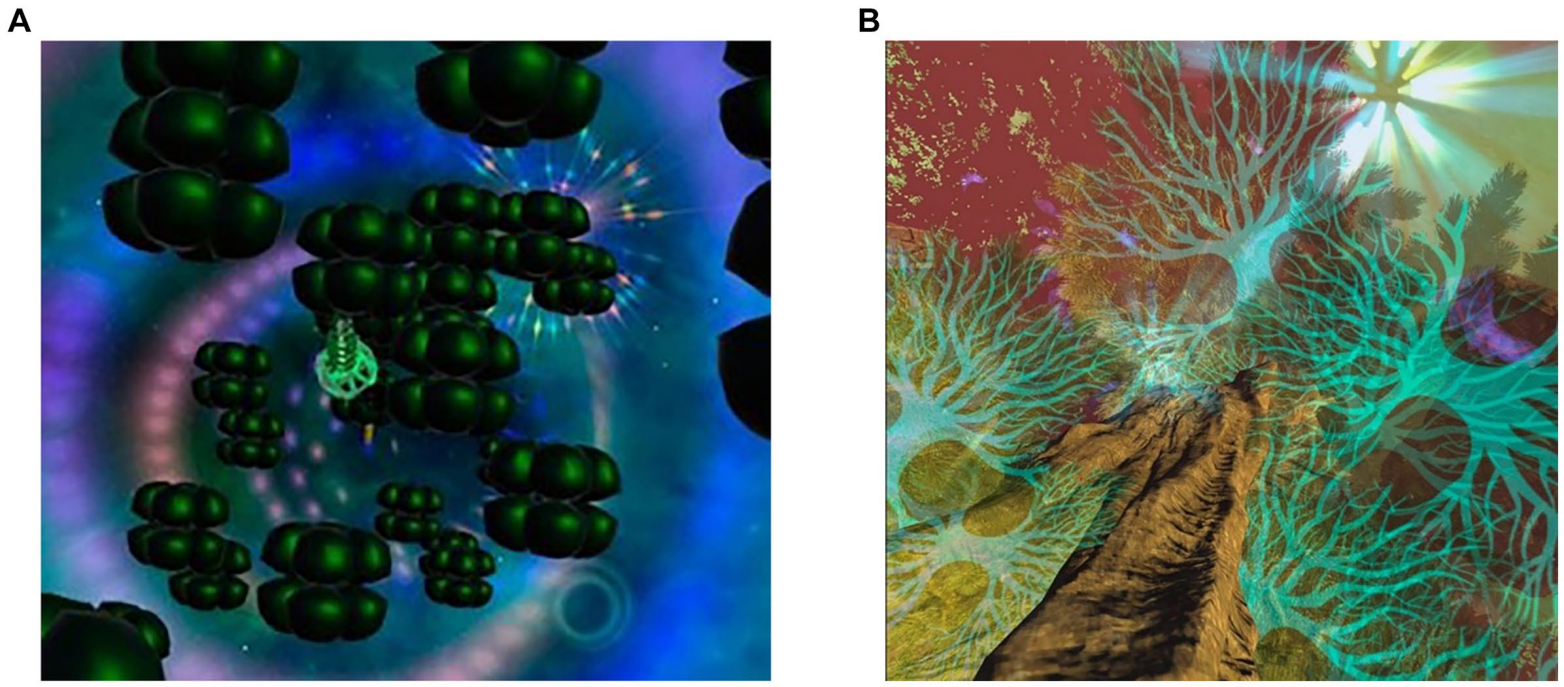
TRIPP active and passive content scenes **(A)** Active, **(B)** Passive. Reproduced with permission from TRIPP, Inc, www.tripp.com.

Screen shots of the dynamic, nature-based visuals in the TRIPP app are depicted in Figures 1A–D, while the active vs. passive screen shots are shown in Figures 2A,B.

### 3.4 Procedure

The study involved the analysis of existing archival data from the consumer VR app TRIPP. Data was composed of de-identified account information, session information, and run information as well as various data associated with each run as allowed by company privacy policy. Users were limited to those accounts reporting as U.S.-based, which TRIPP noted represented about 64% of the accounts available in the dataset. We selected only those worldscape runs with a disposition categorized as either “aborted” or “completed.” An aborted run is indicated when a user engages in worldscape content but does not finish either the entire worldscape experience and/or the mood rating questionnaire at the end. A completed run means the user finished the worldscape experience and also filled out the survey at the end of the run.

### 3.5 Analysis

The method for H1 and H2 was a within-subjects pretest-posttest design comparing reported mood scores as well as usage behavior based on frequency of runs Pre-Pandemic and Pandemic for users of the TRIPP app. Mood Scale scores and frequency of user worldscape runs were analyzed via archival data from 7/24/2019 to 9/1/2020 for US users on the TRIPP VR app. Scores during the COVID-19 event transition period (from and to dates) were discarded from the analysis. Mood Scale score before and after app use was also analyzed to examine mood score change.

The method for H3 was a within-subjects design comparing the Mood Scale score before and after app use to explore mood score change from the active vs. the passive content conditions for both Pre-Pandemic and Pandemic.

## 4 Results

For H1 and H2, data included 181,496 U.S. worldscape runs within 14,653 user accounts. For H3, a smaller sample of 121,140 U.S. worldscape runs categorized as either passive or active was analyzed. Other content outside of these categories was not analyzed.

The natural consumer behavior for users of this particular VR app appeared to be sporadic, decreasing dramatically from the first week of use to later weeks. Therefore, usage behavior for the app content often varied within different time frames, making date and certain time comparisons impractical.

Several variables were extracted and created from the raw data to address the hypotheses.

### 4.1 H1 users will use content more frequently during the pandemic than pre-pandemic

To compare the number of runs Pre-Pandemic and Pandemic, the number of runs per month from November 2019 to June 2020 was examined. This time frame was chosen to give good representation of frequency of use before COVID-19 and after COVID-19, which was defined as before March 9, 2020 and after March 15, 2020, respectively. Additional variables were created that indicated the number of “completed” and “aborted” runs each month from November to June, the number of accounts with a first run each month from November to June, and how many runs were initiated in the first week of use. Preliminary analyses indicated a dramatic drop-off in use for the second week of use, so the first week was chosen as an indication of use Pre-Pandemic and during the Pandemic.

**Figure 3:**
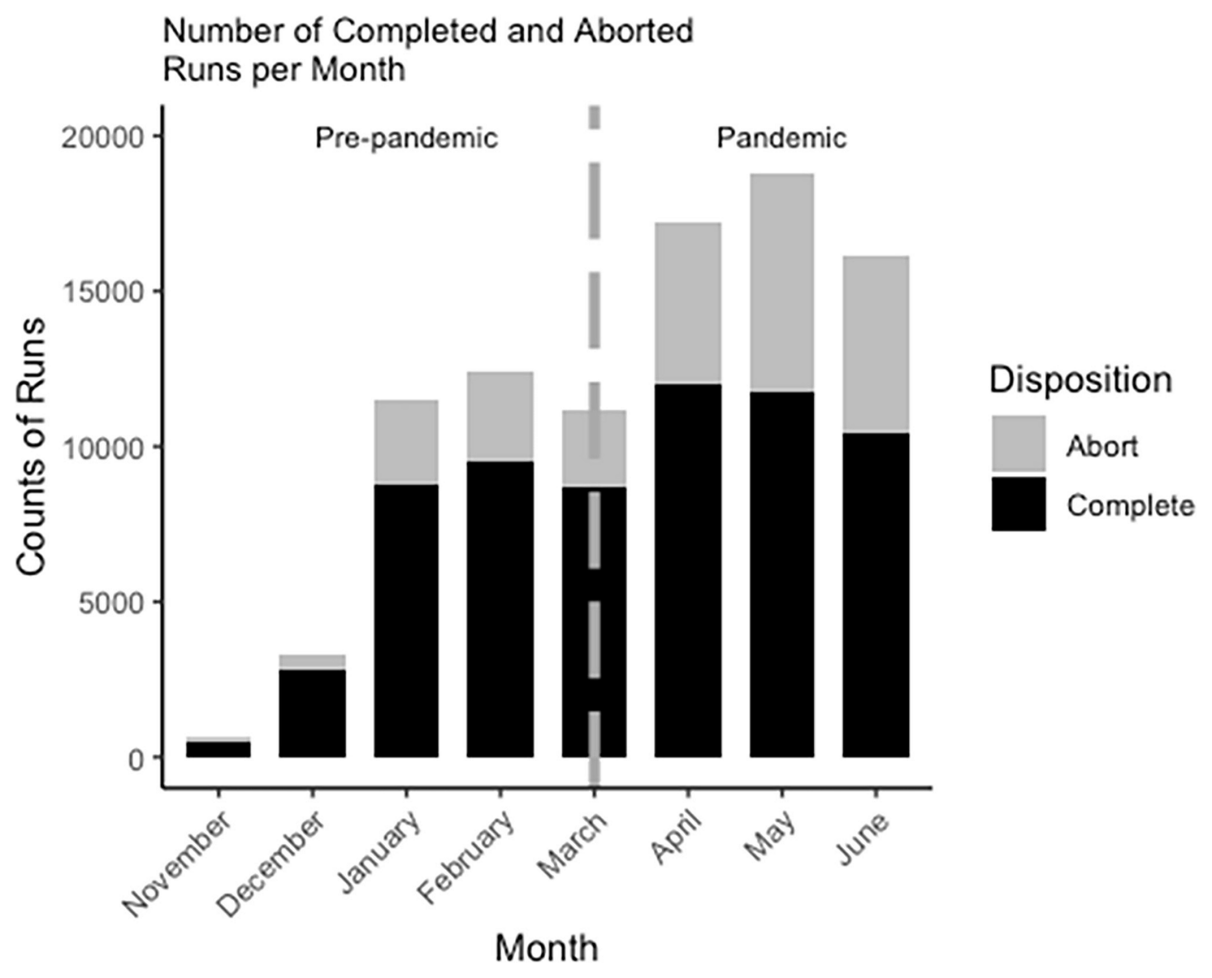
Stacked bar chart of runs per month November to June.

Analyses consisted of frequency counts and is shown in Table 1 by number of runs per month (total, completed, and aborted) as well as the number of accounts with a first run each month. This was illustrated by bar graphs in Figure 3 depicting the number of runs (total, completed, and aborted) each month.

**Table 1:** Number of runs per month.

Month	Aborted runs	Completed runs	Total runs	Accounts with first run
November	88	523	611	50
December	442	2,821	3,263	754
January	2,744	8,752	11,496	1,280
February	2,896	9,545	12,441	874
March	2,500	8,695	11,195	727
April	5,254	11,980	17,234	1,502
May	6,987	11,787	18,774	1,556
June	5,709	10,436	16,145	1,146

The analysis showed a fairly steady number of runs January through March of 2020, and then a sharp increase for April through June after COVID-19. Of additional note is the dramatic increase from December to January, which we speculate is due to holiday-related sales of the TRIPP application. There were more runs of the app during the 3 months after COVID-19 than the 3 months before COVID-19.

**Figure 4:**
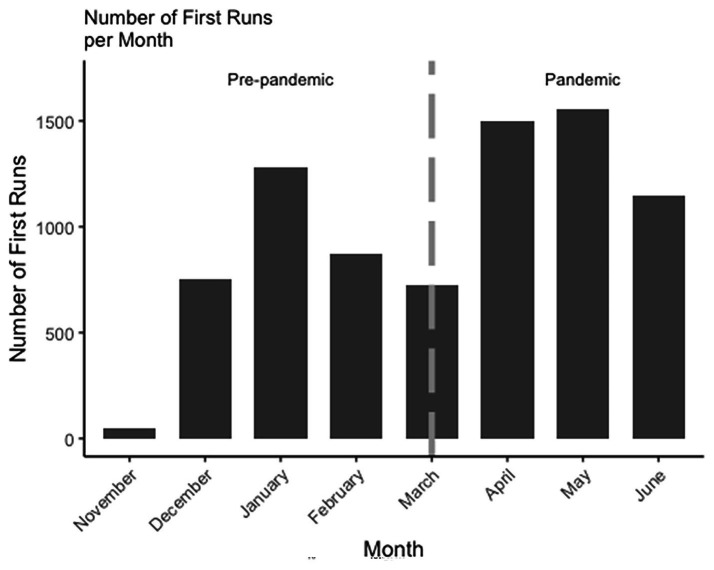
Number of accounts that had a first run each month.

Figure 4 displays the number of accounts that had a first run each month. As is shown, the number of user accounts with first worldscape runs nearly doubled from March to April. Therefore, the jump in number of runs is likely due to the increase in the number of these first time runs after COVID-19.

To see whether there was a change in the number of runs within the first week of use for each account before and after COVID-19, 14,423 accounts were examined. This is slightly less than from the initial dataset, as there were accounts that had an initial session between the COVID-19 dates defined (between 3/9/2020 and 3/15/2020) that were not included.

As Table 2 depicts, *T*-tests of Pre-Pandemic and Pandemic showed that the number of runs the first week of use increased significantly if the first session was after COVID-19.

**Table 2:** *T*-test comparison of number of runs in first week pre-pandemic and pandemic.

	Pre-pandemic	Pandemic	Mean difference	*t*-value	Cohen’s *d*
First week runs	5.78 (5.39)	6.28 (5.60)	0.50 [0.29, 0.72]	4.64***	0.09 [0.05, 0.13]

*p < 0.05, **p < 0.01, ***p < 0.001.

**Figure 5:**
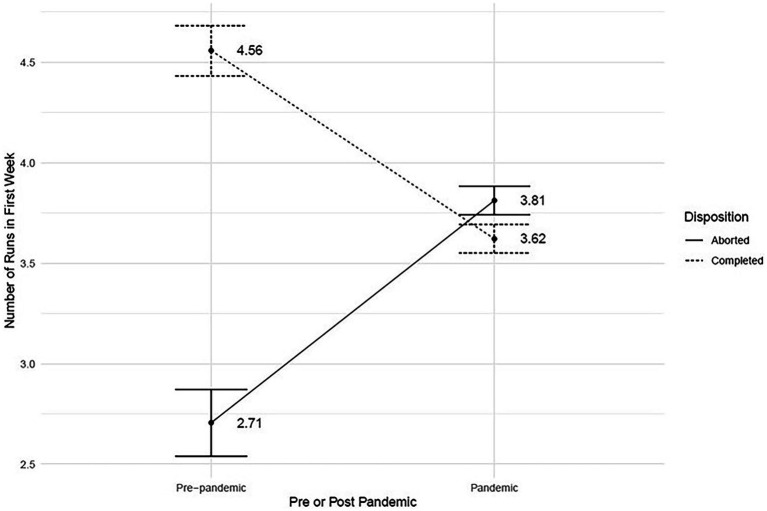
Number of runs by disposition pre-pandemic and pandemic.

In order to investigate if aborted or completed status of runs interacted with the number of first week runs Pre-Pandemic and Pandemic, a 2 × 2 ANOVA was used to compare the number of runs in the first week of use for both Pre-Pandemic and Pandemic. Findings showed that the average number of first week runs per account that were aborted were 2.71 (SD = 2.44) Pre-Pandemic and 3.81 (SD = 3.45) during Pandemic. The average number of first week runs per account that were completed were 4.56 (SD = 4.19) Pre-Pandemic and 3.62 (SD = 3.53) during the Pandemic. The interaction of the 2 × 2 ANOVA was significant *F*(1, 23, 620) = 299.34, *p* < 0.001, partial η^2^ = 0.01. The interaction plot below in Figure 5 depicts that the number of aborted runs increased after COVID-19, and the number of completed runs decreased. All simple effects comparisons between groups were significant.

Next, we calculated and compared the number of user worldscape runs for the week prior and the week after COVID-19 as well as the number of active accounts that had at least one run the week prior and the week after COVID-19. This would provide additional information on usage trends. For the week prior to COVID-19, there were 2,584 worldscape runs and 648 active accounts. For the week right after the start of COVID-19, there were 4,727 worldscape runs and 914 active accounts. This data added to the general trend of more active accounts and more runs after the COVID-19 pandemic.

Finally, we examined changes in use after the COVID-19 onset for 648 accounts that had an active account showing at least one worldscape run before the start of the COVID-19 pandemic. A paired sample t-test indicated the number of runs decreased somewhat in these accounts after COVID-19 as displayed in Table 3.

**Table 3:** Number of runs week prior and week after COVID-19 within accounts.

	Week prior to COVID-19	Week after COVID-19	Mean difference	*t*-value	Cohen’s *d*
Number of runs	3.99 (3.86)	3.20 (5.89)	.-0.79 [−0.38, −1.19]	3.08***	0.15 [0.07, 0.23]

*p < 0.05, **p < 0.01, ***p < 0.001.

### 4.2 H2 users will report lower mood scores at the start of content runs during the pandemic than pre-pandemic

To compare opening mood Pre-Pandemic and Pandemic, a variable was created for the average mood per week within an account. Average mood per week per account provided a more accurate index of mood and eliminated the necessity of equal time periods Pre-Pandemic and Pandemic. A change in mood score was calculated subtracting opening score from the closing score for the mood questionnaires for each run, averaged per week per account.

A paired samples analysis compared opening mood and change in mood scores for those accounts that had at least one run for both Pre-Pandemic and Pandemic. This is shown in Table 4.

**Table 4:** Paired samples *T*-test pre-pandemic pandemic for opening mood and mood change.

	Pre-pandemic	Pandemic	Mean difference	*t*-value	Cohen’s *d*
Opening mood (*n* = 1,187)	5.97 (1.30)	5.76 (1.60)	−0.21 (1.51)	−4.75***	−0.14 [−0.08, −0.19]
Mood change (*n* = 1,103)	1.48 (0.98)	1.56 (1.32)	0.08	2.09*	0.06 [0.004, 0.12]

*p < 0.05, **p < 0.01, ***p < 0.001.

The paired samples *t*-test analysis indicates that mean opening mood significantly decreased from Pre-Pandemic to Pandemic and change of mood had a slight but significant increase. Users reported lower opening mood after the COVID-19 pandemic became a national emergency than before, and the change in mood after engaging in worldscape runs on the app was greater after the pandemic than before. However, it is worth noting that the effect sizes are small for mean mood (*d* = 0.14) and mood score change (*d* = 0.06), and the sample on which the statistical significance is based is large (>1,000).

### 4.3 H3 interactivity level (active vs. passive) will interact with time (pre-pandemic vs. pandemic) such that mood score change will be higher for active content during but not before the pandemic

Worldscape runs were identified as either active with a high level of interaction or passive with a low level of interaction based on the worldscape experience content name and whether it involved an interactive game or not. To see whether content type affected mood score change, intraindividual effects were not examined due to limited available repeated data within accounts, and instead each run, whether active or passive, was treated as an independent event. Mood change variables were created to evaluate change in mood before and after using the VR app content, along with variables to identify whether the worldscape run was Pre-Pandemic (before 03/09/2020) or Pandemic (after 03/15/2020).

Two analyses were conducted to examine the question of how mood change varied between passive and active runs and whether this change varied from Pre-Pandemic and Pandemic. A preliminary independent *t*-test analysis was conducted to see if there was a significant difference in mood change when comparing passive and active runs. The second analysis used a 2 × 2 ANOVA to examine if this change varied Pre-Pandemic and Pandemic.

There were fewer passive runs (*n* = 55,730) than active runs (*n* = 68,410) in this data set. Mean mood change for before and after runs is displayed below in Table 5.

**Table 5:** Mean mood change pre-pandemic and pandemic.

	Total sample	Pre-pandemic	Pandemic	Mean difference
Passive	1.49 (1.52)	1.45 (1.48)	1.51 (1.53)	0.06 [0.02, 0.10]***
Active	1.45 (1.47)	1.46 (1.53)	1.44 (1.45)	−0.02 [−0.05, 0.01]
Difference	0.05*** [0.03, 0.07]	0.01 [0.03, −0.05]	−0.07*** [−0.09, −0.05]	

*p < 0.05, **p < 0.01, ***p < 0.001.

**Figure 6:**
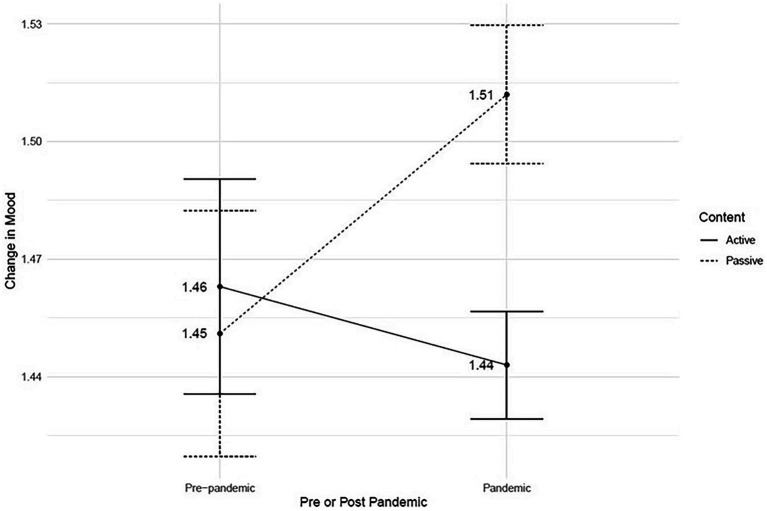
Mood Change and Type of Run Pre- and Post-Pandemic.

For the total sample, the passive runs had a greater mood change than the active runs, but this change was quite small (Cohen’s *d* = 0.03). In addition, there was a significant interaction between type of run (passive or active) and when the run occurred, Pre-Pandemic or Pandemic, when examining mood change, as illustrated in the graph below (Figure 6).

Before COVID-19, there was no difference in effect between passive and active runs in this dataset. After COVID, the passive runs had an effect roughly 1/10 point greater than the active runs. As indicated in the figure above (Figure 6), the difference between active and passive is significant for after COVID-19, but not before COVID-19. The difference between before COVID-19 and after COVID-19 was significant for the passive runs but not for the active runs. It is important to note that while these results are significant, the differences are small.

## 5 Discussion

This study explored how COVID-19 affected behavior and mood for consumer users of a VR app with dynamic content and various levels of interactivity. Analyses addressed three primary questions. First, how did the frequency of use for a VR app with dynamic, interactive content change Pre-Pandemic to Pandemic? How did the reported opening mood and change in mood when using this VR app content vary Pre-Pandemic to Pandemic? Finally, did mood change differ between passive and active worldscape runs and did this change vary from Pre-Pandemic and Pandemic?

### 5.1 COVID-19 onset as predictor for frequency of app content use

A pattern of app use emerged from the analysis findings, which showed increases in use during the COVID-19 pandemic. The total number of worldscape runs per month spiked from April to May as did the total number of accounts with a first run in the data, likely explaining the increase in the number of worldscape runs as new users chose to use the app content to a greater degree shortly after the effects of the COVID-19 pandemic manifested. Likewise, the number of runs for a user’s first week of use increased after COVID-19 became a national emergency. New users in the data used the app more than they did before. Furthermore, the number of runs for the immediate week prior and after COVID-19 increased, coinciding with the start of the pandemic, and active accounts with at least one worldscape run prior and after the pandemic increased as well. The trend in this frequency data all suggest that users created more accounts and used this app content more often after the start of COVID-19.

However, two other relevant findings were discovered during the analysis. To further examine usage of these worldscape runs, such as whether the runs were partially or completely finished from beginning mood survey to closing mood survey, data were examined to compare the frequency of completed or aborted runs. The results showed that there were a greater average number of aborted disposition runs in the first week of use after COVID-19 compared to before COVID, with the opposite pattern for completed runs. It is important to note that a disposition of aborted simply means the user did not complete the entire worldscape run experience and fill out the survey at the end. This implies that first time users tended to not finish the worldscape experiences and/or surveys more often during the week after the pandemic than the week before.

The other finding of interest is that within active accounts, the number of worldscape runs actually decreased significantly when an analysis was conducted looking at the week prior and the week after COVID-19. Users who used the app at least once in the week before the COVID-19 pandemic used it less the week following the COVID-19 pandemic being declared. This indicates that the majority of the increases in accounts and runs were not from active users. However, it is important to consider these results in the context of the trend in the data for each user to generally drop usage from Week 1 to 2. An analysis of whether this decline was to a greater or lesser degree than before the COVID-19 pandemic was not feasible. It is also worth noting that additional weeks for these users were not analyzed to indicate whether this pattern continued as the pandemic progressed.

The findings in this study add to the prior research, in that new or less active users seemed to be more motivated to use the app content more frequently and to complete the worldscape runs, perhaps as a coping mechanism to deal with the added stressors of COVID-19 as suggested by the trends in the data after the start of COVID-19. This appears to be consistent with theories of supportive design, arousal, mood management, stress and coping, as well as self-regulation in the choice of content (Berlyne, 1971; Folkman, 1984; Zillmann, 1988a,b; Bandura, 1991; Ulrich et al., 1991; Ulrich, 2001).

However, users appeared to abort runs more frequently and users who used the app in the week before COVID-19 did not appear to use it as much in the week after COVID-19. Based on the literature, there could be various reasons for this. The uses and gratifications (U&G) theory, along with the compensatory internet use theory, suggest that users are motivated to use devices and features of those devices in order to satisfy an array of psychological needs, including tension-release, diversion, affective or aesthetic experience needs, and emotional regulation (Kardefelt-Winther, 2014; Rauschnabel, 2018). This suggests that users may have been able to achieve self-regulation and the appropriate level of arousal during COVID-19 with less runs, thus relieving stress and improving mood prior to completing the experience. Users were motivated to use the app only for the duration of time it took to achieve the desired psychological benefits, including distraction from outside stressors related to the pandemic.

Of course, another possibility along these lines is that users who were initially motivated to use the app content lost motivation to start and continue runs because the content was not meeting these increased demands during the COVID-19 pandemic. Interestingly, if this is the case, then this could actually be good news for app developers because it indicates that during “normal” everyday life outside of a pandemic, users were more motivated to use the app content to meet their psychological needs.

Finally, because there was a general trend for users to drop off usage from the first week of use in the data to the second week, it is possible that lack of interest with the worldscape experiences and/or surveys may have influenced users to abort or stop using the app before the run or the survey was completed over time. It is also possible that the app content did not adequately meet some user’s needs and that these users would have chosen to engage in the content for less time on Week 2 regardless of COVID-19.

Features of the VR device itself might also play a role in this as well because immersion in VR requires disengaging from the external environment, which may be difficult to maintain repeatedly, particularly for those who are very busy or require frequent awareness of their surroundings. This would have been particularly relevant with users at home during the pandemic, potentially with children, pets, or other people around.

It is possible that interest in use for new users was initially very high as COVID-19 pandemic accounts skyrocketed with users seeking new ways to cope with the increased stressors but that interest tapered out over time as users were unable to get the long-term stress relief they were seeking. Additional longitudinal studies would be informative.

### 5.2 COVID-19 as predictor for mood scores

This study compared mood scores and mood change before and after COVID-19, with findings indicating that users reported lower opening mood scores after COVID-19 started than before it was declared a national emergency in the US and greater positive changes in mood after engaging in worldscape runs on the app as well. When individual user mood scores were compared, there was a slight decrease in opening mood as well as a slight positive increase of change in mood from Pre-Pandemic to Pandemic. This appears to agree with the literature that suggests reported mood may be reflective of a more generalized outlook and persistent affective state than other briefer emotional states (Lazarus, 1991; Morris, 1992; Russell, 2003, 2005; Ekkekakis, 2013). These findings, along with the spike in accounts, runs, and frequency of use for H1, indicates that users may have chosen to enter the worldscape experiences in order to self-regulate these increased negative mood states. Indeed, device content that is able to create positive emotions and improve mood is expected to motivate user behavior (Fredrickson, 2001).

Additionally, this analysis shows that the change in mood scores from opening to closing was higher after these users engaged in worldscape runs than it was before the pandemic, despite overall mood being lower prior to using the app. This demonstrates that the VR app content may function as a feature of the environment to meet users’ psychological needs during increased stress and successfully distract users from negative stimuli, even improving mood states during the pandemic. Immersive elements, sense of presence, interactivity, and aesthetic content features of a VR app may all influence the level of stress relief as well as user perceptions of control and positive distraction (Ulrich et al., 1991; Lessiter et al., 2001; Schubert et al., 2001; Ulrich, 2001; Freeman, 2004; De Kort et al., 2006; Gerber et al., 2017; Tanja-Dijkstra et al., 2018). Unfortunately, measures of these factors were not available to better elucidate which contributed to a greater extent to improve mood, but some interplay between them is very likely based on the prior literature.

### 5.3 COVID-19 as predictor for interactivity level usage and mood

Because of the unique properties of highly interactive content, it was predicted that a more active level of interactive content would result in higher mood score change than a more passive level of interactive content during COVID-19. If users feel that needs for control, positive distraction, tension-release, diversion, affective or aesthetic are met, or if mood is improved, users should continue to be motivated to use that content, while experiencing stress reduction (Ulrich et al., 1991; Fredrickson, 2001; Ulrich, 2001; Kardefelt-Winther, 2014; Rauschnabel, 2018). Content that produces the greatest sense of presence and distraction from outside environmental stressors, such as those that offer high levels of interactivity, should be exceptionally effective (Witmer and Singer, 1998).

Surprisingly, the opposite pattern emerged than that which was predicted based on the literature. Specifically, users chose to use more interactive content in general, but the less interactive content was slightly more effective for enhancing reported mood following these worldscape experiences during COVID-19. Both levels of interactivity appeared to improve mood, but the less interactive, more calming content led to slightly higher positive mood score changes only after the start of the COVID-19 pandemic. Because users still preferred to use the more interactive content with higher levels of complexity across the studied time frame, despite its lower mood score effects after COVID-19 onset, this suggests that the stress-reducing and psychological effects of content with more interactivity may be better measured via some other short-term affective state, while less interactive content designed to be more restorative may have a stronger effect on overall mood during stressful events.

While interactivity influences presence, other factors such as positive distraction may have a more important role for reducing stress, creating a sense of restoration, and improving mood. The increased technological strain and overstimulation from working and going to school virtually with regular interaction requirements during the pandemic may have also partly influenced these findings. A more passive experience could be more effective at restoration and mood improvement by reducing cognitive and performance demands. However, because interactive content has been shown to be more fun and create a greater sense of presence and perceived control, users may have chosen to use it more and derive benefits from it that were not measured in this study. It may have influenced a more temporal affective state or perhaps the mechanism by which active levels of interactivity effects stress is more related to pleasant levels of arousal within the rewards circuit that alleviates boredom or intrusive thoughts. The challenges of higher levels of interactivity may be more motivating and the rewards greater when the user achieves some goal. Other measures of emotional states, arousal levels, escapism, and experiences of pleasure, tension-release, or hedonic value may better elucidate this relationship.

It is important to note that some of the effect sizes were small in this study (e.g., for H3, mood change between active and passive run was Cohen’s *d* = 0.03). However, the results seem to follow a general trend for these hypotheses that reveals COVID-19 had an effect on user behavior and mood states and that specific content may have had more influence than others for this effect.

## 6 Limitations

Consumer archival data may present unique challenges for the researcher in that the collection, analysis, and interpretation for findings to research questions may have to adapt to accommodate the available data that were collected. Certain method modifications may be needed to address unexpected data, the appearance of inconsistencies, and/or limitations discovered during analysis. In this study, for example, we had to be creative in how to approach measuring sporadic data values that did not nicely match up with one another on a timeline. In these situations, open communication between those involved in development with the app and the researcher is imperative.

Another issue is the accuracy of collected data and researcher interpretation. Consumer data may involve an abundance of unknowns for the researcher since it is not possible to observe users at home inputting the data or control for the multitude of factors and variables that may play a role in any analysis results. It is not known, for example, whether an account in this study was actually used only by one individual or by multiple individuals. Along the same lines, is the question of how representative survey responses reported on a consumer app may actually be for any particular user. Reporting reliable demographic data for this study was particularly challenging since users may choose not to provide voluntary information or may input incorrect data. For example, user age could not be ascertained on any analysis due to reliability concerns since a number of users reported an unreasonably high age in years (>100).

Researchers should be cautious when using large consumer data sets to support interpretations for significant findings. Although a large *N* provides higher external validity due to generalization, and reduces selection bias, significant results may be discovered due to the high number of participants in the sample. Since *p*-values depend upon both the magnitude of association and the sample size, *p*-values can be considered significant at *p* < 0.05, even if the magnitude of effect is small. Therefore, significant findings, which may be interesting, may not be as helpful for predicting the effects of an intervention. Looking at data trends may be a more informative approach with large sample sizes, particularly to compare research questions around data that extend over a period of time as well as before and after an event or intervention. It is also possible that although a large sample can provide some generalizability, the fact that an entire consumer dataset that met certain criteria for one specific app was analyzed without randomization may make these results difficult to apply to users of any other VR app, let alone users of technology in general, or the general population during a pandemic.

While the single mood score measure appears to have good face validity as a subjective measure for mood within subjects, other measures of validity were not conducted. Additionally, reliability of the measure has not been established. Further research would be useful in exploring whether a one question numerical mood measure correlates with more established mood measures.

It is also worth noting that our selection for the time frame of the COVID-19 intervention revolved around the March 11 date when COVID-19 was declared a pandemic by the WHO and the March 13, 2020 date when COVID-19 was declared a national emergency in the US with a few days cushion in-between. Certainly, users may have been aware of the issue prior to these dates or may have been slower to get an understanding of the evolving situation and its direct effect on them. Therefore, usage behavior or mood may have been impacted earlier or later than the dates we used to describe the COVID-19 intervention.

We look forward to future research to begin to untangle the threads.

## 7 Conclusion

This study sought to show whether a relationship appeared to exist between COVID-19, behavior, and mood for users of a VR app with dynamic, interactive content as well as promote additional research that may build upon this topic. After examining archival user data for a VR app with dynamic, interactive content before and after the COVID-19 crisis, our statistical analysis concluded that the COVID-19 pandemic had significant effects on user behavior and mood.

The majority of users in the dataset used content more frequently during COVID-19 than before. Users did report lower mood scores at the start of using app content during COVID-19 than before, suggesting that the pandemic had a negative effect on mood. Additionally, mood appears to have positively increased to a greater degree when users engaged in the VR app after the pandemic started, making this app an effective mood booster, even with the increasing stressors related to COVID-19. Other study findings contradict the prior literature around interactivity and expected findings of superior stress reduction and mood improvement. Results of this study show that a more passive level of interactive content improved mood to a greater degree after the start of the COVID-19 pandemic, although users did choose to engage in more active forms of interactive content regardless of COVID-19 pandemic status. This indicates that the passive content was likely more restorative for users, positively increasing mood, but that the more active content also likely provided some psychological effect that motivated users to continue to engage in it.

The implications of these findings indicate that users outside of a controlled environment are likely to seek out VR apps with the potential to regulate stress in their everyday lives during increased psychosocial stressors, such as a pandemic. Additionally, measures of mood collected from consumer archival data may adequately capture related increases in stress and negative affect, while VR apps with dynamic, interactive content can improve wellbeing measures, such as mood, even with reported declines. Furthermore, the benefit of the content type used by consumers may vary for differing psychological needs. In this case, passive content may improve overall mood more effectively during increasingly stressful events, and thus be the most useful intervention to employ during pandemic-like contexts. However, the relationship between pandemic stressors, usage behavior, mood, and the existing research is not yet definitive.

While we conclude that brief mood measures such as that used in this study may be an appropriate measure for users experiencing pandemic stress, additional measures of stress and affective states may better indicate this relationship. Additionally, it is possible that other types of VR app content, which users continue to find engaging over time, could produce stronger effects. Future research should build on our understanding of longitudinal pandemic stress, its effects on users, and what consumer devices and content may be most useful to reduce that stress in the home environment. A better understanding of features related to certain content and devices that contribute to stress reduction and improved mood is paramount in order to develop effective resources for those with restrictions or limited access to normal coping mechanisms.

## Data availability statement

The data analyzed in this study is subject to the following licenses/restrictions: the data analyzed in this study was obtained from TRIPP. TRIPP will not authorize the public release of this data because it is proprietary and trade secret. Other researches who wish to use this data to validate or build upon our findings can contact research@trippinc.com to obtain a copy of this anonymous dataset. Requests to access these datasets should be directed to research@trippinc.com.

## Ethics statement

The studies involving humans were approved by Fielding Graduate University IRB. The studies were conducted in accordance with the local legislation and institutional requirements. Written informed consent for participation was not required from the participants or the participants’ legal guardians/next of kin in accordance with the national legislation and institutional requirements.

## Author contributions

JH: Conceptualization, Data curation, Formal analysis, Investigation, Methodology, Writing – original draft, Writing – review & editing. AC: Formal analysis, Methodology, Supervision, Writing – review & editing. KS: Supervision, Writing – review & editing.

## References

[ref1] American Psychological Association. (2020). Stress in America 2020: A national mental health crisis.

[ref2] Bandura A. (1991). Social cognitive theory of self-regulation. Organ. Behav. Hum. Decis. Process. 50, 248–287. doi: 10.1016/0749-5978(91)90022-L

[ref3] Berlyne D. E. (1971). Aesthetics and psychobiology. New York: Appleton-Century-Crofts.

[ref4] Boylan P. Kirwan G. H. Rooney B. (2018). Self-reported discomfort when using commercially targeted virtual reality equipment in discomfort distraction. Virtual Reality 22, 309–314. doi: 10.1007/s10055-017-0329-9

[ref5] Burns-Nadera S. Joe L. Pinion K. (2017). Computer tablet distraction reduces pain and anxiety in pediatric burn patients undergoing hydrotherapy: a randomized trial. Burns 43, 1203–1211. doi: 10.1016/j.burns.2017.02.015, PMID: 28318748

[ref6] Centers for Disease Control and Prevention. (2020). Mental health household pulse survey. Available at: https://www.cdc.gov/nchs/covid19/pulse/mental-health.htm (Accessed June 10, 2020).

[ref7] Centers for Disease Control and Prevention. (2023). Trends in number of COVID-19 cases and deaths in the US reported to CDC, by state/territory. Available at: https://covid.cdc.gov/covid-data-tracker/#trends_totaldeaths_select_00 (Accessed January 29, 2023).

[ref8] De Kort Y. Meijnders A. L. Sponselee A. A. G. Ijsselsteijn W. A. (2006). What's wrong with virtual trees? Restoring from stress in a mediated environment. J. Environ. Psychol. 26, 309–320. doi: 10.1016/j.jenvp.2006.09.001

[ref9] Drigas A. Mitsea E. (2020). A metacognition based 8 pillars mindfulness model and training strategies. Int. J. Recent Contributions Eng. Sci. 8, 4–17. doi: 10.3991/ijes.v8i4.17419

[ref9002] Drigas A. Mitsea E. Skianis C. (2022). Virtual Reality and Metacognition Training Techniques for Learning Disabilities. Sustainability, 14:10170.

[ref10] Ekkekakis P. (2013). The measurement of affect, mood, and emotion: a guide for health-behavioral research. New York: Cambridge University Press.

[ref11] Fan Y. Pestke K. Feeser M. Aust S. Pruessner J. C. Böker H.. (2015). Amygdala–hippocampal connectivity changes during acute psychosocial stress: joint effect of early life stress and oxytocin. Neuropsychopharmacology 40, 2736–2744. doi: 10.1038/npp.2015.123, PMID: 25924202 PMC4864649

[ref12] Folkman S. (1984). Personal control and stress and coping processes: a theoretical analysis. J. Pers. Soc. Psychol. 46, 839–852. doi: 10.1037/0022-3514.46.4.839 6737195

[ref13] Fredrickson B. L. (2001). The role of positive emotions in positive psychology: the broaden-and-build theory of positive emotions. Am. Psychol. 56, 218–226. doi: 10.1037/0003-066X.56.3.218 11315248 PMC3122271

[ref14] Freeman J. (2004). Implications for the measurement of presence from convergent evidence on the structure of presence. In Paper presented at the May 2004 conference of the international communication association, New Orleans, LA

[ref15] Gerber S. M. Jeitziner M. Wyss P. Chesham A. Urwyler P. Müri R. M.. (2017). Visuo-acoustic stimulation that helps you to relax: a virtual reality setup for patients in the intensive care unit. Sci. Rep. 7, 13228–13210. doi: 10.1038/s41598-017-13153-1, PMID: 29038450 PMC5643433

[ref16] Hoffman H. G. Meyer W. J. Ramirez M. Roberts L. Seibel E. J. Atzori B.. (2014). Feasibility of articulated arm mounted oculus rift virtual reality goggles for adjunctive pain control during occupational therapy in pediatric burn patients. Cyberpsychol. Behav. Soc. Netw. 17, 397–401. doi: 10.1089/cyber.2014.0058, PMID: 24892204 PMC4043256

[ref17] Hoffman H. G. Patterson D. R. Seibel E. Soltani M. Jewett-Leahy L. Sharar S. R. (2008). Virtual reality pain control during burn wound debridement in the hydrotank. Clin. J. Pain 24, 299–304. doi: 10.1097/AJP.0b013e318164d2cc, PMID: 18427228

[ref18] Hoffman H. G. Rodriguez R. A. Gonzalez M. Bernardy M. Peña R. Beck W.. (2019). Immersive virtual reality as an adjunctive non-opioid analgesic for pre-dominantly Latin American children with large severe burn wounds during burn wound cleaning in the intensive care unit: A pilot study. Front. Hum. Neurosci. 13:262. doi: 10.3389/fnhum.2019.00262, PMID: 31440148 PMC6694842

[ref19] Hoffman H. G. Seibel E. J. Richards T. L. Furness T. A. Patterson D. R. Sharar S. R. (2006). Virtual reality helmet display quality influences the magnitude of virtual reality analgesia. J. Pain 7, 843–850. doi: 10.1016/j.jpain.2006.04.006, PMID: 17074626

[ref20] Housand J. (2021). Effect of the COVID-19 pandemic on psychological stress and usage behavior for users of a VR app with dynamic, interactive content. Order no. 28720004. Doctoral dissertation, fielding graduate university. ProQuest Dissertations & Thesis Global.

[ref21] Kardefelt-Winther D. (2014). A conceptual and methodological critique of internet addiction research: towards a model of compensatory internet use. Comput. Hum. Behav. 31, 351–354. doi: 10.1016/j.chb.2013.10.059

[ref22] Katz E. Blumler J. G. Gurevitch M. (1973). Uses and gratifications research. Public Opin. Q. 37, 509–523. doi: 10.1086/268109

[ref9001] Katz E. Blumler J. G. Gurevitch M. (1974). Ulilization of mass communication by the individual. In Blumler J. G. Katz E. (Eds.), The uses of mass communications: Current perspectives on gratifications research 19–32. Beverly Hills: Sage.

[ref23] Kelly R. M. Seabrook E. M. Foley F. Thomas N. Nedeljkovic M. Wadley G. (2022). Design considerations for supporting mindfulness in virtual reality. Front. Virtual Real. 2:672556. doi: 10.3389/frvir.2020.00001

[ref24] Khadra C. Ballard A. Déry J. Paquin D. Fortin J. S. Perreault I.. (2018). Projector-based virtual reality dome environment for procedural pain and anxiety in young children with burn injuries: A pilot study. J. Pain Res. 11, 343–353. doi: 10.2147/JPR.S151084, PMID: 29491717 PMC5817417

[ref25] Lazarus R. S. (1991). Emotion and adaptation. New York: Oxford University Press.

[ref26] Leger K. A. Charles S. T. Almeida D. M. (2018). Let it go: lingering negative affect in response to daily stressors is associated with physical health years later. Psychol. Sci. 29, 1283–1290. doi: 10.1177/0956797618763097, PMID: 29553880 PMC6088503

[ref27] Lessiter J. Freeman J. Keogh E. Davidoff J. (2001). A cross-media presence questionnaire: the ITC-sense of presence inventory. Presence Teleop. Virt. 10, 282–297. doi: 10.1162/105474601300343612

[ref28] Maani C. V. Hoffman H. G. Morrow M. Maiers A. Gaylord K. McGhee L. L.. (2011). Virtual reality pain control during burn wound debridement of combat related burn injuries using robot-like arm mounted VR goggles. J. Trauma 71, S125–S130. doi: 10.1097/TA.0b013e31822192e2, PMID: 21795888 PMC4460976

[ref29] Mazgelyte E. Rekiene V. Dereskeviciute E. Petrenas T. Songailiene J. Utkus A.. (2021). Effects of virtual reality-based relaxation techniques on psychological, physiological, and biochemical stress indicators. Healthcare 9:1729. doi: 10.3390/healthcare9121729 34946455 PMC8701384

[ref30] Mitsea E. Drigas A. Charalampos S. (2022). Mindfulness for anxiety management and happiness: the role of VR, metacognition, and hormones. Technium BioChemMed. 3, 37–52. doi: 10.47577/biochemmed.v3i3.7343

[ref31] Mitsea E. Drigas A. Skianis C. (2023). VR gaming for meta-skills training in special education: the role of metacognition, motivations, and emotional intelligence. Educ. Sci. 13:639. doi: 10.3390/educsci13070639

[ref32] Morris W. N. (1992). “A functional analysis of the role of mood in affective systems” in Review of personality and social psychology. ed. Clark M. S., vol. 13 (Sage), 256–293.

[ref33] Piskorz J. Czub M. (2018). Effectiveness of a virtual reality intervention to minimize pediatric stress and pain intensity during venipuncture. J. Specialists Pediatric Nursing 23:e12201. doi: 10.1111/jspn.12201, PMID: 29155488

[ref34] Rauschnabel P. A. (2018). Virtually enhancing the real world with holograms: an exploration of expected gratifications of using augmented reality smart glasses. Psychol. Mark. 35, 557–572. doi: 10.1002/mar.21106

[ref35] Rubin A.M. (2002). The uses-and-gratifications perspective of media effects. Hillsdale, NJ: Erlbaum.

[ref36] Russell J. A. (2003). Core affect and the psychological construction of emotion. Psychol. Rev. 110, 145–172. doi: 10.1037/0033-295X.110.1.145 12529060

[ref37] Russell J. A. (2005). Emotion in human consciousness is built on core affect. J. Conscious. Stud. 12, 26–42.

[ref38] Schubert T. Friedmann F. Regenbrecht H. (2001). The experience of presence: factor analytic insights. Presence Teleop. Virt. 10, 266–281. doi: 10.1162/105474601300343603

[ref39] Seabrook E. Kelly R. Foley F. Theiler S. Thomas N. Wadley G.. (2020). Understanding how virtual reality can support mindfulness practice: mixed methods study. J. Med. Internet Res. 22:e16106. doi: 10.2196/16106, PMID: 32186519 PMC7113800

[ref40] Segerstrom S. C. Miller G. E. (2004). Psychological stress and the human immune system: A meta-analytic study of 30 years of inquiry. Psychol. Bull. 130, 601–630. doi: 10.1037/0033-2909.130.4.601, PMID: 15250815 PMC1361287

[ref41] Sil S. Dahlquist L. M. Thompson C. Hahn A. Herbert L. Wohlheiter K.. (2014). The effects of coping style on virtual reality enhanced videogame distraction in children undergoing cold pressor pain. J. Behav. Med. 37, 156–165. doi: 10.1007/s10865-012-9479-0, PMID: 23184062

[ref42] Small C. Stone R. Pilsbury J. Bowden M. Bion J. (2015). Virtual restorative environment therapy as an adjunct to pain control during burn dressing changes: study protocol for a randomised controlled trial. Trials 16:329. doi: 10.1186/s13063-015-0878-8, PMID: 26242401 PMC4526294

[ref43] Soltani M. Dreyer S. Hoffman H. Sharar S. Wiechman S. Jensen M.. (2018). Virtual reality analgesia for burn joint flexibility: A randomized controlled trial. Rehabil. Psychol. 63, 487–494. doi: 10.1037/rep0000239, PMID: 30284865 PMC6235624

[ref44] Song H. Fang F. Amberg F. K. Mataix-Cols D. Fernández de la Cruz L. Almqvist C.. (2019). Stress related disorders and subsequent risk of life threatening infections: population based sibling controlled cohort study. BMJ 367:l5784. doi: 10.1136/bmj.l5784, PMID: 31645334 PMC6812608

[ref45] Spiegel B. Fuller G. Lopez M. Dupuy T. Noah B. Howard A.. (2019). Virtual reality for management of pain in hospitalized patients: A randomized comparative effectiveness trial. PLoS One 14:e0219115. doi: 10.1371/journal.pone.0219115, PMID: 31412029 PMC6693733

[ref46] Tanja-Dijkstra K. Pahl S. White M. P. Auvrey M. Stone R. J. Andrade J.. (2018). The soothing sea: A virtual coastal walk can reduce experienced and recollected pain. Environ. Behav. 50, 599–625. doi: 10.1177/0013916517710077, PMID: 29899576 PMC5992839

[ref47] Ulrich R. (2001). “Effects of healthcare environmental design on medical outcomes” in Design and health. ed. Delani A. (AB: Flanders Svenski Truck).

[ref48] Ulrich R. S. Simons R. F. Losito B. D. Fiorito E. Miles M. A. Zelson M. (1991). Stress recovery during exposure to natural and urban environments. J. Environ. Psychol. 11, 201–230. doi: 10.1016/S0272-4944(05)80184-7

[ref49] Witmer B. G. Singer M. J. (1998). Measuring presence in virtual environments: A presence questionnaire. Presence Teleop. Virt. 7, 225–240. doi: 10.1162/105474698565686

[ref50] Zillmann D. (1988a). Mood management through communication choices. Am. Behav. Sci. 31, 327–340. doi: 10.1177/000276488031003005

[ref51] Zillmann D. (1988b). “Mood management: using entertainment to full advantage” in Communication, social cognition, and affect. eds. Donohew L. Sypher H. E. Higgins E. T. (Hillsdale, NJ: Lawrence Erlbaum Associate), 147–171.

[ref52] Zillmann D. (2000). Mood management in the context of selective exposure theory. Ann. Int. Commun. Assoc. 23, 103–123. doi: 10.1080/23808985.2000.11678971

