# Supplementary material for: Greater usage and positive mood change for users of a dynamic VR app before and after the COVID-19 pandemic onset
Source: Front Psychol. 2024 Feb 27;15:1278207. doi: 10.3389/fpsyg.2024.1278207 (PMC10929007; doi:10.3389/fpsyg.2024.1278207)
Supplement: Supplementary file 1 [file Data_Sheet_1.docx]

Supplementary Material

Greater Usage and Positive Mood Change for Users of a Dynamic VR App Before and After the COVID-19 Pandemic Onset

Jessica Housand*, Allen Cornelius, Karen E. Shackleford

*** Correspondence:** Jessica Housand: jhousan1@jh.edu

# Supplementary Video to Figure 2

TRIPP Active and Passive Content Scenes Video Samples

Fig2**(A)** <https://drive.google.com/file/d/1sM0asm32n2ghNR4apn8qKePI1xEagc24/view?usp=sharing>

Fig2**(B)** <https://youtu.be/53dK_ExV0YY>

**
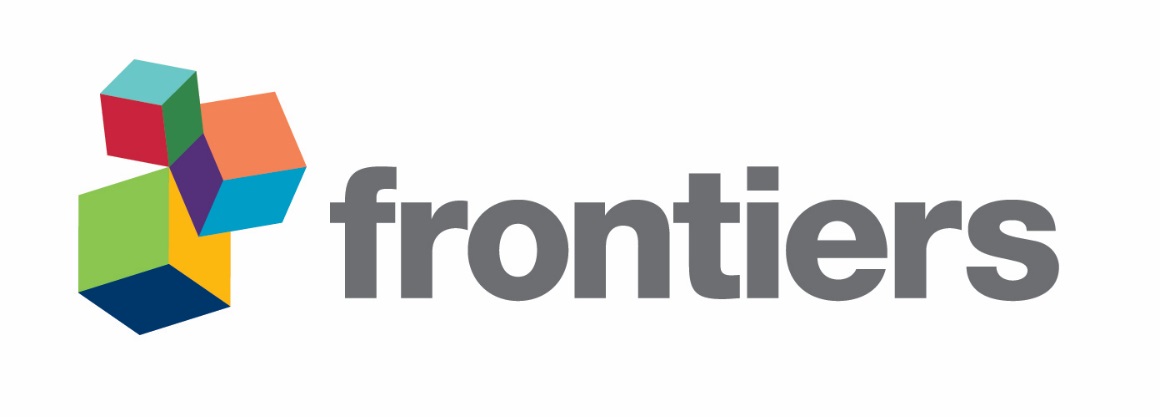
**
